# Nanoindentation Response of 3D Printed PEGDA Hydrogels in a Hydrated Environment

**DOI:** 10.1021/acsapm.2c01700

**Published:** 2023-01-20

**Authors:** Mohammad Hakim Khalili, Craig J. Williams, Christian Micallef, Fabian Duarte-Martinez, Ashfaq Afsar, Rujing Zhang, Sandra Wilson, Eleftheria Dossi, Susan A. Impey, Saurav Goel, Adrianus Indrat Aria

**Affiliations:** † Surface Engineering and Precision Centre, School of Aerospace, Transport and Manufacturing, Cranfield University, Cranfield MK43 0AL, U.K.; ‡ The Henry Royce Institute, Department of Materials, The University of Manchester, Manchester M13 9PL, U.K.; § School of Chemistry, University of Edinburgh, David Brewster Road, Edinburgh EH9 3FJ, U.K.; ∥ Centre for Defence Chemistry, Cranfield University, Shrivenham, Swindon SN6 8LA, U.K.; ⊥ Sophion Bioscience A/S, Baltorpvej 154, 2750 Ballerup, Denmark; # London South Bank University, 103 Borough Road, London SE1 0AA, U.K.; ∇ University of Petroleum and Energy Studies, Dehradun 248007, India

**Keywords:** poly(ethylene glycol) diacrylate, nanoindentation, 3D printing, cross-linked hydrogels, creep behavior

## Abstract

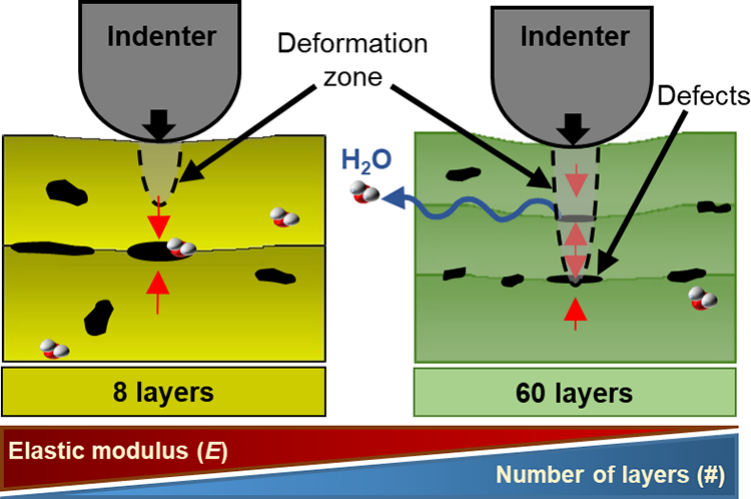

Hydrogels are commonly used materials in tissue engineering and organ-on-chip devices. This study investigated the nanomechanical properties of monolithic and multilayered poly(ethylene glycol) diacrylate (PEGDA) hydrogels manufactured using bulk polymerization and layer-by-layer projection lithography processes, respectively. An increase in the number of layers (or reduction in layer thickness) from 1 to 8 and further to 60 results in a reduction in the elastic modulus from 5.53 to 1.69 and further to 0.67 MPa, respectively. It was found that a decrease in the number of layers induces a lower creep index (C_IT_) in three-dimensional (3D) printed PEGDA hydrogels. This reduction is attributed to mesoscale imperfections that appear as pockets of voids at the interfaces of the multilayered hydrogels attributed to localized regions of unreacted prepolymers, resulting in variations in defect density in the samples examined. An increase in the degree of cross-linking introduced by a higher dosage of ultraviolet (UV) exposure leads to a higher elastic modulus. This implies that the elastic modulus and creep behavior of hydrogels are governed and influenced by the degree of cross-linking and defect density of the layers and interfaces. These findings can guide an optimal manufacturing pathway to obtain the desirable nanomechanical properties in 3D printed PEGDA hydrogels, critical for the performance of living cells and tissues, which can be engineered through control of the fabrication parameters.

## 1 Introduction

Engineered hydrogel bioscaffolds have been extensively used in the area of biomedical engineering including drug delivery and tissue engineering due to their biocompatibility and the ability to tune their mechanical properties.^1−4^ Additive manufacturing, such as three-dimensional (3D) printing, of bioscaffolds offers the ability to customize morphological and mechanical properties for specific tissue engineering applications such as cell encapsulations and 3D tissue formation.^5−8^ A large body of literature reports that cell behaviors, including growth, migration, proliferation, differentiation, and tissue formation are all strongly influenced by mechanical cues at the substrate interface on which the cells are cultured.^9−11^ For instance, the fate of stem cells can be directed by engineering the surface elastic modulus, topography, and adhesion of the hydrogel substrates.^12−14^ The cell elongation direction and sarcomere alignment of muscle tissues can also be influenced by the surface elastic modulus of hydrogel substrates.^15−18^ A projection lithography 3D printing technique enables rapid fabrication of photo-cross-linkable hydrogels with complex geometrical features, e.g., high aspect ratio pillars, lattices, or overhangs, and an alteration in associated mechanical characteristics.^19,20^ This makes it critical to fully understand the surface mechanical properties of hydrogels for designing and engineering smarter bioscaffold structures expanding functionality.^21−23^ A previous study has demonstrated the contractile force measurement of muscle tissue strips using 3D printed poly(ethylene glycol) diacrylate (PEGDA) hydrogel cantilevers,^24^ underpinned by the ability to engineer the elastic modulus and selective adhesion at the interface between the tissue strip and the PEGDA cantilever.

While bulk mechanical measurements such as uniaxial compression testing and flexural bending tests provide useful information about the mechanical properties of 3D printed PEGDA structures,^7,24−26^ they are unsuitable for assessing the spatial variation in surface mechanical properties critical for cell adhesion and tissue formation.^27−29^ Moreover, bulk measurement on 3D printed structures with complex geometry and anisotropic characteristics, e.g., high aspect ratio and multilayered,^20^ is nontrivial to ensure precision alignment of the applied force vector to the principal axes of the layers.^30^ Such misalignment can lead to imprecise measurements from buckling or inhomogeneous surface loading and maybe more pronounced in multilayered structures with layer thickness in sub-tens of microns.^30^ In contrast, methods such as nanoindentation and atomic force microscopy (AFM) are more suitable for assessing the surface properties of multilayered 3D printed structures,^27,31,32^ particularly when the alignment between the applied force and layer orientation is critical.^33−37^

In this study, the effect of layer number and thickness on nanomechanical and creep behavior of multilayered PEGDA hydrogel surfaces printed by layer-by-layer (LbL) projection lithography was investigated. It is demonstrated herein that the surface elastic modulus increases from 0.67 to 5.53 MPa by increasing the layer thickness from 20 to 1200 μm. This finding is attributed to structural imperfections introduced by LbL fabrication using projection lithography and is in contrast to the extant understanding of the mechanical behavior of 3D multilayered hydrogels with the common conception of “the thinner the stronger”.^38−40^ This new understanding is critical for predicting the functionality of 3D printed PEGDA hydrogels, which are of great interest in biomedical applications, as their nanomechanical characteristics may significantly affect the behavior and performance of living cells and tissues.

## 2 Materials and Methods

### 2.1 Printing of PEGDA 3D Structures Using Projection Lithography

A detailed description of methods of hydrogel sample printing, glass treatment, and drying has been described in a previous work.^20^ The aqueous prepolymer solution contained 200 mg/mL poly(ethylene glycol) diacrylate (PEGDA, *M*_n_ 700 g/mol) and 5 mg/mL photoinitiator lithium phenyl-2,4,6-trimethylbenzoylphosphinate (LAP, ≥95%). For LbL printing of multilayered PEGDA hydrogels with variable layer thickness (multi-20 μm and multi-150 μm), two prepolymer solutions with addition of 9 and 1.8 mg/mL quinoline yellow (QY) photoabsorber were prepared, respectively. An ultraviolet (UV) light source (wavelength of 365 nm) with an intensity of 20 mW/cm^2^ was used. Each layer was exposed for 3 or 15 s, depending on the required dosage of either 120 or 600 mJ/cm^2^, respectively, to ensure cross-linking of every layer of the multilayer structure using a custom-made automated projection lithographic printer.

### 2.2 Multilayered Samples

**Figure 1:**
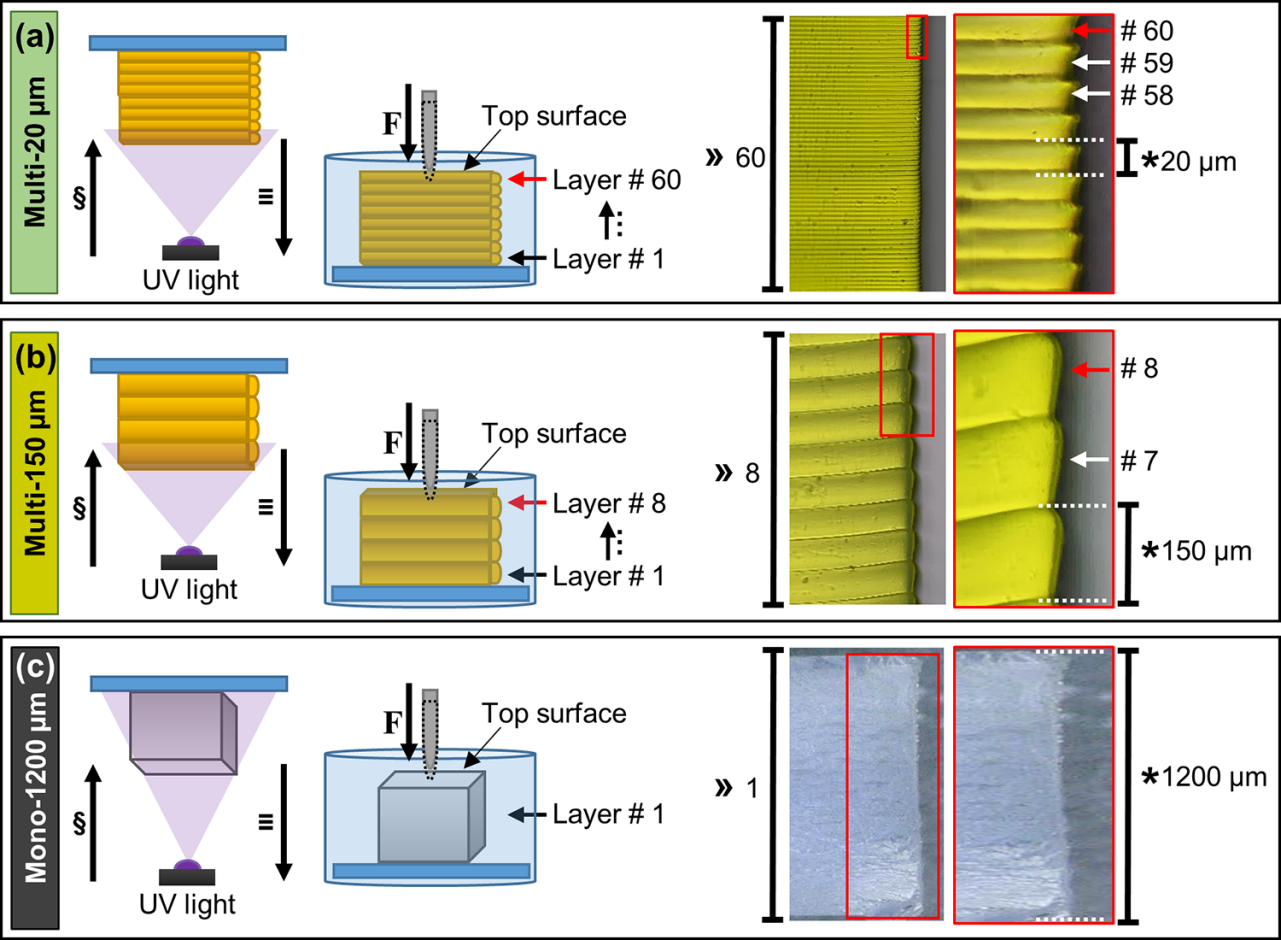
Schematics (left) of the light and print direction and (right) side-view optical microscopy photographs of (a) multi-20 μm, (b) multi-150 μm, and (c) mono-1200 μm PEGDA hydrogel structures. Samples stored in deionized water (DIW) and cell culture medium (CCM) at room temperature (20–21 °C) at a predefined storage time points (0–720 h after fabrication) before indentation. (§) Projection light direction, (≡) printing direction, (F) indentation direction, (≫) total number of layers, (*) individual layer thickness. Note that the total structural height for all samples was 1200 μm.

All multilayered samples were printed on a 15 mm diameter laser cut and surface-treated glass slide to fit the well of a nanoindentation liquid cell. The cuboid multilayered sample dimensions were 5 × 5 × 1.2 mm^3^ (*L* × *W* × *H*). To maintain a sample thickness of 1.2 mm, samples with 20 μm layer thickness consisting of 60 layers and samples with 150 μm layer thickness consisting of eight layers were prepared. Hence, the number of layers was inversely proportional to the nominal layer thickness. The samples were printed using LbL projection lithography with a 3 s UV exposure time. The samples were labeled herein as multi-20 μm-3 s and multi-150 μm-3 s (Figure 1a,b). The printed samples were stored in either deionized (DI) water (DIW, resistivity of 18.2 MΩ·cm) or cell culture medium (CCM) for 24 and 720 h at room temperatures between 20 and 21 °C. The CCM (Gibco RPMI 1640 Medium, Fisher scientific, Loughborough, U.K., with l-glutamine) is a standard cell culture medium, which uses a sodium bicarbonate buffer system of 2 g/L for in vitro diagnostic use.^41^ Additionally, for investigating the effect of the number of layers on the mechanical properties, multilayered hydrogel samples with four and 32 layers were fabricated, labeled multi-300 μm-3 s and multi-37.5 μm-3 s, respectively. The QY photoabsorber concentration in the hydrogel samples, multi-300 μm-3 s and multi-37.5 μm-3 s, was kept the same as the sample multi-150 μm-3 s at 1.8 mg/mL in the prepolymer solution. This eliminated the effect of variation in the QY photoabsorber concentration in the hydrogels produced. Moreover, a set of multilayered samples with a 15 s UV exposure time with 20 and 150 μm layers was printed and named multi-20 μm-15 s and multi-150 μm-15 s, respectively (Table 1). The samples were stored in DIW for 24 h before indentation measurements.

**Table 1:** List of Multilayered and Monolithic Samples Printed for the Nanoindentation Measurements in This Study^a^

sample name	QY (mg/mL)	# of layers	layer thickness (μm)	UV exposure time (s)	UV dosage (mJ/cm^2^)
multi-20 μm-3 s	9	60	20	3	120
multi-20 μm-15 s	9	60	20	15	600
multi-150 μm-3 s	1.8	8	150	3	120
multi-150 μm-15 s	1.8	8	150	15	600
multi-300 μm-3 s	1.8	4	300	3	120
multi-37.5 μm-3 s	1.8	32	37.5	3	120
mono-1200 μm-3 s	0	1	1200	3	120
mono-1200 μm-15 s	0	1	1200	15	600

a The overall structure height was maintained at 1.2 mm for all samples.

### 2.3 Monolithic Structures

For single-layer monolithic samples, a prepolymer solution of PEGDA and LAP was poured into a vat, and the sample stage was moved to a height of 1200 μm. Cuboid monolithic structures of dimensions 5 × 5 × 1.2 mm^3^ (*L* × *W* × *H*) were fabricated with UV exposure times of 3 and 15 s, labeled mono-1200 μm-3 s and mono-1200 μm-15 s, respectively (Table 1). The fabricated samples were then stored in DI water for 24 h before measurements (Figure 1c). The hydrogel structures were fabricated on the surface of a treated microscope glass slide (76 × 26 × 1–1.2 mm^3^, Fisher Scientific, Loughborough, U.K.) cut to 24 × 24 × 1–1.2 mm^3^ to fit the sample holder.

### 2.4 Nanomechanical Measurements

Nanoindentation experiments were carried out using a Hysitron Bioindenter (Bruker Hysitron, Minneapolis) fitted with a 50 μm diameter, spherical diamond tip. Samples were mounted on coverslip glasses and fixed to a standard glass slide. Tests were carried out in a hydrated environment using a custom-designed liquid cell that fits over a glass slide to create a well for the solution. PEGDA hydrogels were stored in DIW and CCM for 24 and 720 h before testing. After mounting the hydrogel in the liquid cell inside the machine’s enclosure, the sample was left for at least 2 h to reach equilibrium to minimize any effect of thermal drift. The indentations were carried out in a load-controlled manner with a maximum load of up to 20 μN. The load profile of 5 s load, 2 s hold period, and 5 s unload segments was used. For each sample, 12 indents were performed at random locations on top of the hydrogel samples. The indenter was set with a 2 μN preload to ensure that the tip was in contact with the sample surface.^42^ The surface of the sample in contact with the probe is the final layer, top surface, exposed to UV light during printing (Figure 1). The samples were analyzed by extracting *P*–*h* (Figure S1) curves from the Hysitron Triboscan Analysis software, and the elastic modulus (*E*) was estimated using the Oliver and Pharr method by analyzing the unloading curve of the nanoindentation curve using eqs S1–S6. For quantifying the creep behavior, the normalized simple isothermal creep index (C_IT_) obtained from eq S7 and creep strain rate (ε̇) calculated using eq S8(43,44) were used.

### 2.5 Gravimetric Measurements

In a previous study, the thermal response of 3D multilayered PEGDA hydrogels was investigated through a gravimetric method for samples stored in deionized water (DIW).^20^ As 3D PEGDA hydrogels are intended for use in biological applications, their thermal response was expanded to include the hydrogel stored in cell culture medium (CCM) baths at 8, 20, and 37 °C. Two cuboid samples of multi-20 μm (comprising of 255 layers) and two multi-150 μm (comprising of 34 layers) PEGDA hydrogels with dimensions 5 × 5 × 5.10 mm^3^ were fabricated, using 3 s UV exposure time, for each temperature in DIW and CCM baths.

**Figure 2:**
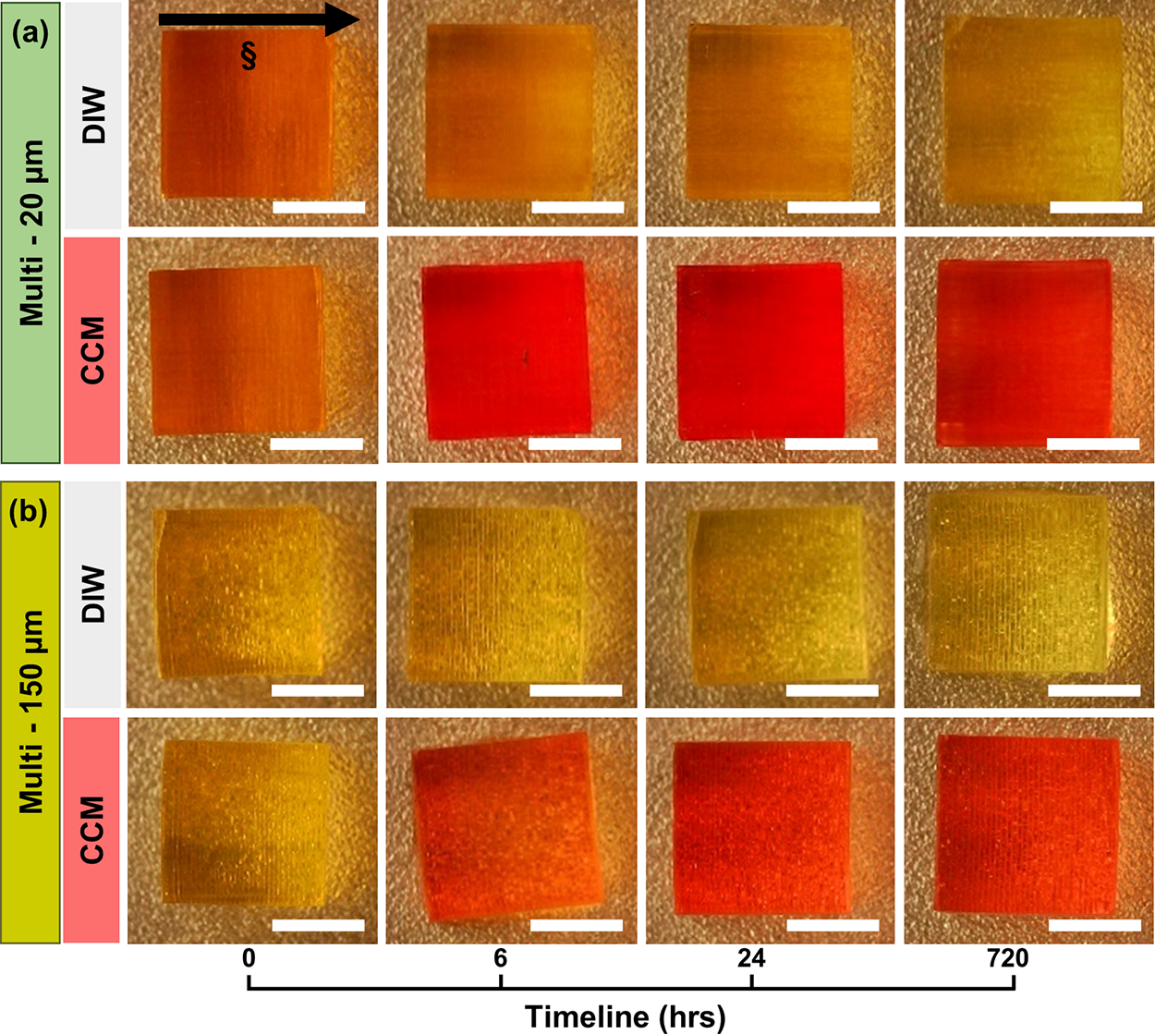
Side-view photographs of 3D multilayered (a) multi-20 μm-3 s and (b) multi-150 μm-3 s PEGDA hydrogel samples stored in DIW (upper) and CCM (lower) at room temperature at predefined storage time points (0–720 h after fabrication), demonstrating the inward diffusion of CCM into the hydrogel system and outward diffusion of the QY photoabsorber from the hydrogel into the DIW storage solution. Zero hour is the time immediately after printing. The scale bars in all images are 5 mm. Note that the dark shades within the hydrogels are due to uneven illumination of the microscope. Direction of projection light (§) is from top to bottom of the structure.

Immediately after fabrication, 3D multilayered PEGDA hydrogel samples were washed with DIW to remove unreacted prepolymers from the surface, gently blotted using medical wipes, weighed, and placed in their assigned DIW and CCM baths at their controlled-temperature environment (Figure 2). The weight of the hydrogel samples was monitored every 2 h over 6, 24, 48 h, and finally at 720 h (30 days). At each time point, the hydrogel samples were taken out of their storage solution, gently blotted using tissue paper, and weighed before returning to the same storage solution. The samples were vacuum-dried in a desiccator for 24 h at room temperature at the end of 720 h study. Their dried weight (*M*_d_) was measured the following day using an analytical balance.

The normalized weight fraction (NWF) was used to calculate the weight change using eq 1(20)
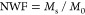
1where (*M*_s_) and (*M*_0_) are the weight of the hydrogel at time *t* and weight of the hydrogel immediately after fabrication, i.e., time 0, respectively.

### 2.6 ^1^H NMR Measurements

All starting materials, PEGDA monomer, LAP photoinitiator, and QY photoabsorber were characterized by proton nuclear magnetic resonance (^1^H NMR) spectroscopy in deuterium oxide (D_2_O; Merck 151882, ≥99.9%, *M*_W_ 20.03 g/mol), at ambient temperature, using a Bruker Ascend 400 MHz spectrometer with a BBFO probe and tetramethylsilane (TMS; Merck 87920, ≥99.5%, *M*_W_ 88.22 g/mol) as an internal standard (Figure S2). The soluble fractions isolated from the swelling experiments of both 3D multilayered hydrogel samples with Ø 5 mm of diameter and 2.5 mm of thickness (samples multi-20 μm-3 s, multi-20 μm-15 s, multi-150 μm-3 s, and multi-150 μm-15 s) and monolithic hydrogel samples (samples mono-1200 μm-3 s and mono-1200 μm-15 s) fabricated using projection lithography at UV exposure times 3 and 15 s were characterized by ^1^H NMR. The procedure to obtain the soluble fractions involved vacuum-drying hydrogel samples immediately after fabrication. They were stored in 7 mL of D_2_O, for 24 h, allowing time for the hydrogel to swell and any residual starting materials to leach out of the structure. The dried residue was then characterized by ^1^H NMR in D_2_O solution.

### 2.7 Differential Scanning Calorimetry (DSC) Measurements

The glass transition temperature (*T*_g_) of the 3D PEGDA hydrogels was determined by differential scanning calorimetry (DSC; Mettler Toledo, DSC 3 + STAR System) using a heating rate of 10 °C min^–1^ under an inert N_2_ atmosphere. The test temperature was cycled twice between −100 and 25 °C. The variation of the heat flow in the samples was recorded as a function of temperature and time. Multilayered hydrogel samples, multi-20 μm-3 s and multi-150 μm-3 s, respectively, with Ø 5 mm of diameter and 2.5 mm of thickness, previously stored in DIW and CCM for 24 and 720 h, were dried and then characterized in triplicate (approximately 9–14 mg). Additionally, multilayered, multi-20 μm-3 s, multi-20 μm-15 s, multi-150 μm-3 s, and multi-150 μm-15 s, and monolithic, mono-1200 μm-3 s and mono-1200 μm-15 s, PEGDA hydrogels exposed to 3 and 15 s UV light, stored in DI water for 24 h, were characterized after being dried.

### 2.8 Scanning Electron Microscopy (SEM)

SEM (VEGA3, TESCAN) at an acceleration voltage of 20 kV was used to image the cross section of vacuum-dried PEGDA hydrogels. The layers of the multilayered hydrogels were exposed by manually cutting the sample vertically along the printing direction using a 100 μm thick stainless steel blade. A 20 nm gold film was deposited on each sample using a sputter coater prior to imaging. The backscattered images were captured at ×150 and ×600 magnifications.

## 3 Results

**Figure 3:**
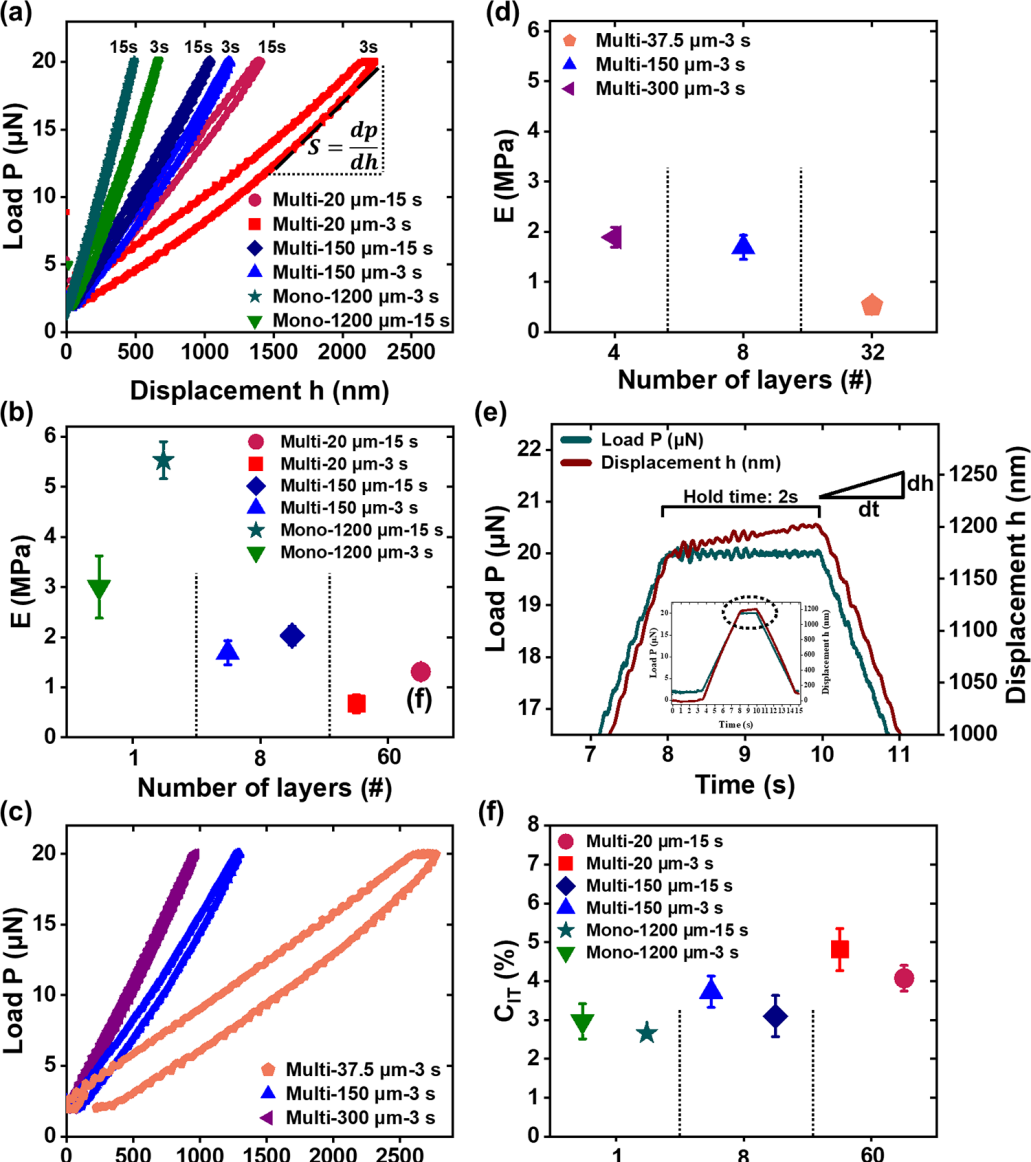
(a, c) Representative *P*–*h* curves and (b, d) elastic modulus (*E*); (f) creep index (C_IT_) obtained from nanoindentation of 3D multilayered PEGDA hydrogels, multi-20 μm-3 s (■), multi-20 μm-15 s (●), multi-150 μm-3 s (▲), multi-150 μm-15 s (**◆**), multi- 300 μm-3 s (◀), and multi-37.5 μm-3 s (⬟) and monolithic hydrogels, mono-1200 μm-3 s (▼) and mono-1200 μm-15 s (★) after 24 h of storage in DIW post printing. (e) (*P*–*h*) curve demonstrates the creep depth profile under a constant load for 2 s. The error bars in panels (b) and (d) represent combined uncertainty of samples *n* = 2 with 12 indents in each *n*, except mono-1200 μm-3 s, mono-1200 μm-15 s, multi-20 μm-15 s, and multi-150 μm-15 s where *n* = 1 with 12 indents for each sample. The error bars in panel (e) represent the standard deviation from a mean of *n* = 3.

As shown in Figure 3a, the (*P*–*h*) plot of indents made on the multi-20 μm-3 s hydrogel revealed a gentle slope of 7.2 N/m that was less than half and less than quarter compared to the plot of multi-150 μm-3 s and mono-1200 μm-3 s hydrogels of 16.1 and 32 N/m, respectively. The highest indentation depth, 2.4 μm was seen on multi-20 μm-3 s, compared to the multi-150 μm-3 s and mono-1200 μm-3 s hydrogels where the indentation depth was about 1.2 and 0.6 μm, respectively. Figure 3b shows that *E* of the multi-20 μm-3 s (60 layers) is approximately 2.5 times lower than that of the multi-150 μm-3 s (8 layers). The decrease in *E* was correlated negatively with the increase in the number of layers or interfaces within the PEGDA hydrogel structures. As shown in Figure 3b, the monolithic PEGDA hydrogel, mono-1200 μm-3 s, had an *E* of 5.53 ± 0.37 MPa that was about 3-fold and 8-fold higher than those of the multi-150 μm-3 s and multi-20 μm-3 s, respectively. Figure 3b also shows that *E* of both multilayered hydrogels increased by (96 and 20%) and *E* in the monolithic hydrogel increased by 84% when the UV exposure time was increased from 3 to 15 s. The increase in UV dosage from 3 to 15 s may lead to an increase in the degree of cross-linking in both multilayered and monolithic PEGDA hydrogels.

To elucidate the effect of the number of layers on the compliance under an indentation area and its immediate surrounding area, additional samples were fabricated and tested with varying number of layers; these include four layers (multi-300 μm-3 s), eight layers (multi-150 μm-3 s), and 32 layers (multi-37.5 μm-3 s). The overall height of all samples was maintained at 1200 μm. The same QY photoabsorber concentration of 1.8 mg/mL was used for all samples. The *P*–*h* curve for these hydrogels (Figure 3c) shows that increasing the number of layers from four layers in multi-300 μm-3 s to 32 layers in multi-37.5 μm-3 s results in a 2-fold increase in displacement. This is equivalent to a decrease in *E* from 1.89 to 0.53 MPa as the number of layers increases from 4 to 32 (Figure 3d).

The effect of increasing the number of layers and UV exposure time was further investigated by analyzing the creep behavior of 3D printed PEGDA hydrogels. During a 2 s holding period, where the indentation load was kept constant, the indentation depth continues to increase, which led to a creep deformation (Figure 3e). The multi-20 μm-3 s PEGDA hydrogels showed the highest average creep displacement of 106 nm, followed by multi-150 μm-3 s and then mono-1200 μm-3 s at 43 and 19 nm, respectively. At the beginning of the load holding period, the penetration increased at a very high strain rate between 0.35 and 0.03 s^–1^, indicating “transient creep” behavior (Figure S3b).^45^ A steady-state period follows, where the strain increases linearly with time and equilibrates at the order of about 0.01 s^–1^, depending on the stiffness of 3D PEGDA hydrogels. The creep index (C_IT_) (eq S7), which quantifies the creep deformation, for multi-20 μm-3 s was 4.8%, approximately 37.5% higher than that for mono-1200 μm-3 s at 3% (Figure 3f). An increase in the UV light exposure time from 3 to 15 s leads to a decrease in C_IT_ of 10–16%.

**Figure 4:**
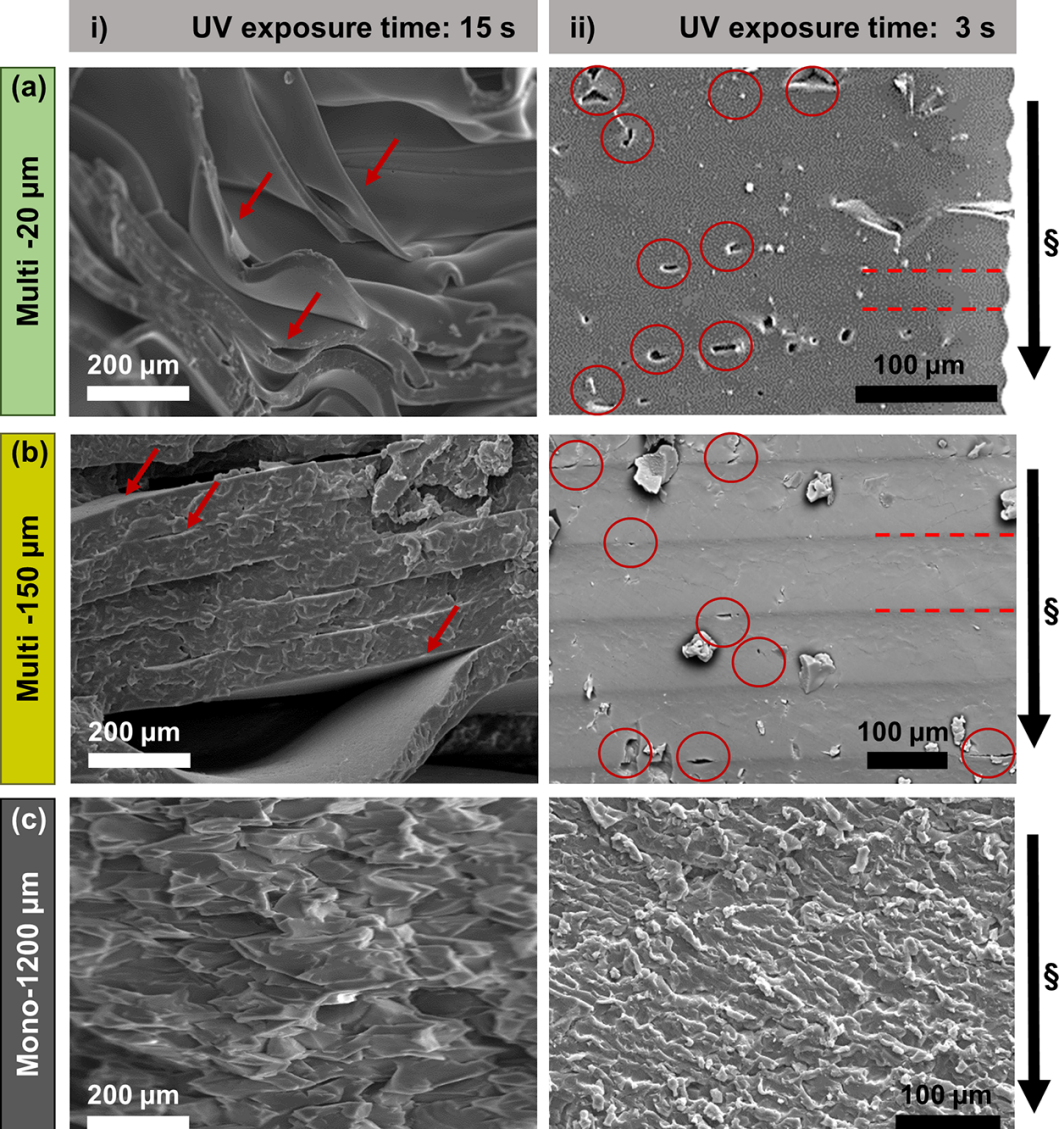
SEM images of cross section of vacuum-dried 3D PEGDA hydrogels: (a) (i) multi-20 μm-15 s and (b) (i) multi-150 μm-15 s showing delamination of layers (left-side images with red arrows) with samples exposed to 15 s of UV light. (a) (ii) Multi-20 μm-s and (b) (ii) multi-150 μm-3 s showing structural imperfections and localized pockets of voids due to unreacted prepolymers (right-side images with red circles) at the interfaces and within the layers with samples exposed to 3 s of UV light. The dark areas are the interfaces of hydrogels (indicated with red dotted lines). (c) (i) Mono-1200 μm-15 s and (ii) mono-1200 μm-3 s PEGDA hydrogels showing a smearing effect due to blade cutting the hydrogel to reveal the cross section. The direction of the projection light (§) is from top to bottom of the structure.

The observed dependency on the layer thickness or number of layers motivated an investigation of the interfaces between layers and defect density using SEM. Cross-sectional SEM images of vacuum-dried multi-150 μm-15 s and multi-20 μm-15 s PEGDA (Figure 4ai,bi) showed delamination of individual layers at multiple locations, indicating weaker interlayer interfaces compared to the hydrogel network within the layer itself. Interface failure could be due to the residual stress at the interface between the top section of the previously printed layer and the bottom section of the newly formed layer. The top section is assumed to be sufficiently cross-linked as it is closer to the UV light, while the bottom section is farther away from the UV light and is assumed to be less cross-linked in comparison.^46,47^ Any inhomogeneity or gradient in cross-linking within each layer is attributed to the absorption of the UV light by the photoabsorber in the prepolymer solution resulting in a spatial decay of light intensity during the cross-linking process.^48,49^ There was no obvious change in the monolithic mono-1200 μm-3 s and mono-1200 μm-15 s in terms of their visual appearance, as there is no interlayer interface within the structure (Figure 4cii, right). High magnification cross-sectional SEM images of vacuum-dried multilayer PEGDA showed imperfections that appear as pockets of voids, both at interfaces and within printed layers.^39^ These structural imperfections (Figure 4aii,bii) are more evident in the multi-20 μm-3 s than in the multi-150 μm-3 s and not apparent in SEM images of monolithic PEGDA hydrogels (Figure 4c).

**Figure 5:**
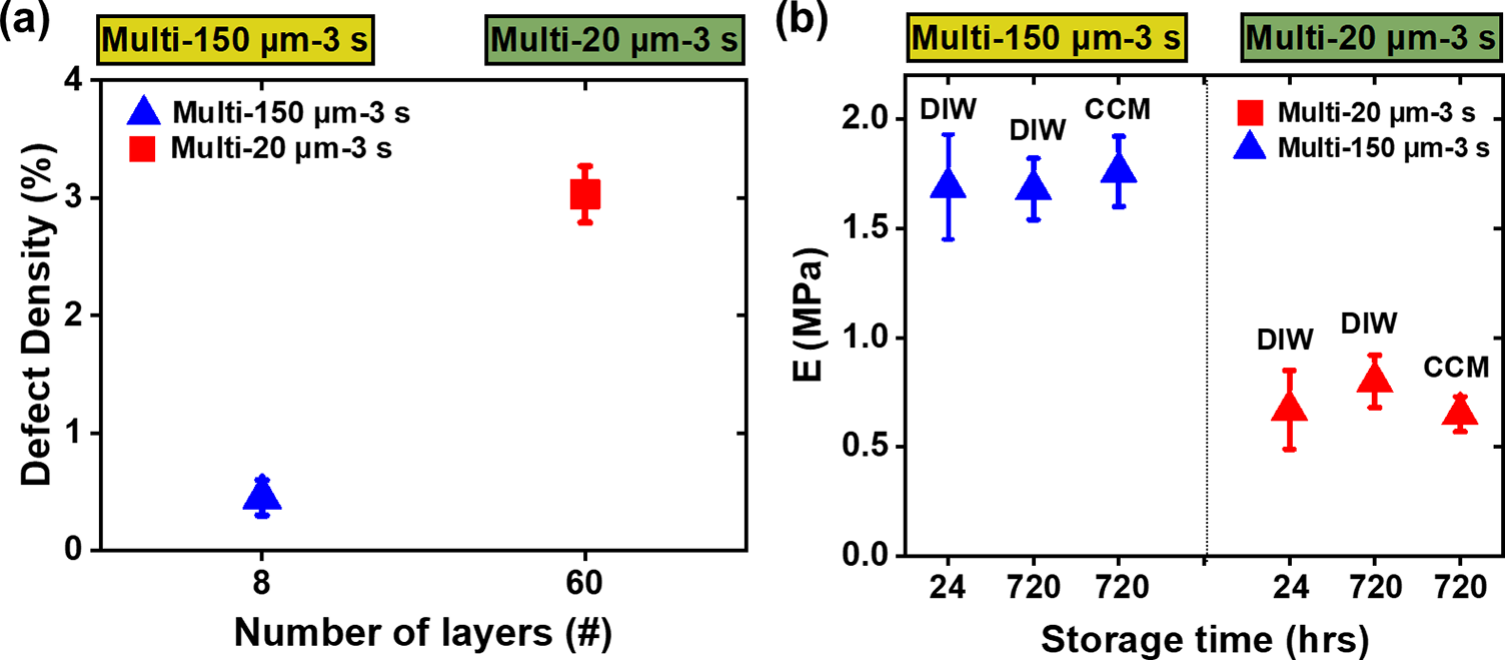
(a) Defect density in 100 μm^2^ for both multi-20 μm (■) and multi-150 μm (▲) PEGDA hydrogel samples based on the measurement of defect areas captured in SEM images (Figure S4). (b) Nanomechanical stability of multilayered PEGDA hydrogels. *E* for both 3D multi-20 μm-3 s (■) and multi-150 μm-3 s (▲) PEGDA hydrogels stored in DIW and CCM for 720 h, compared with samples stored only for 24 h in DIW. The error bars represent (a) uncertainty of samples *n* = 2 and (b) combined uncertainty of samples *n* = 2 with 12 indents in each *n*.

The presence of voids observed in SEM images of the multilayered PEGDA hydrogels was seen to impact the mechanical properties.^50^ In this study, the defect density of multilayered PEGDA structures was approximated by apparent void surface area per unit area assuming uniform distribution in a lateral direction (Figure S4 and Table S2). The defect density was approximated to be almost 7-fold greater in the multi-20 μm-3 s than that in the multi-150 μm-3 s (Figure 5a). This agrees with the negative correlation between *E* and the number of layers (Figure 3b), where the monolithic mono-1200 μm-3 s showed significantly higher *E* compared to the multilayered PEGDA hydrogels with no apparent defects in the dried state.

Storage in DIW and CCM solutions over 720 h had a negligible effect on the mechanical stability of the hydrogel samples (Figure 5b). For 3D multi-20 μm-3 s hydrogels, the average *E* increased by + 20%, from 0.67 ± 0.18 to 0.80 ± 0.12 MPa, when the samples stored in DIW for 720 h compared to the samples stored in DIW for 24 h after fabrication (Figure 5b). For the same hydrogels stored in CCM for 720 h, *E* was constant. For the multi-150 μm-3 s PEGDA, *E* was constant for samples stored both in DIW and CCM for 24 and 720 h (Figure 5b). The absence of correlation between storage time and *E* of multilayered PEGDA suggests that the fabricated hydrogels may have reached equilibrium within 24 h and no further cross-linking or structural modification takes place for 720 h after printing. In agreement with our previous study,^20^ such stability is also reflected by relatively constant gravimetric measurements (Figure S5) and glass transition temperature (*T*_g_) (Figure S6a,b) for 720 h. This suggests that 3D printed PEGDA hydrogels are relatively stable under any fabrication and storage conditions and with negligible effect of layer numbers and thicknesses.

**Figure 6:**
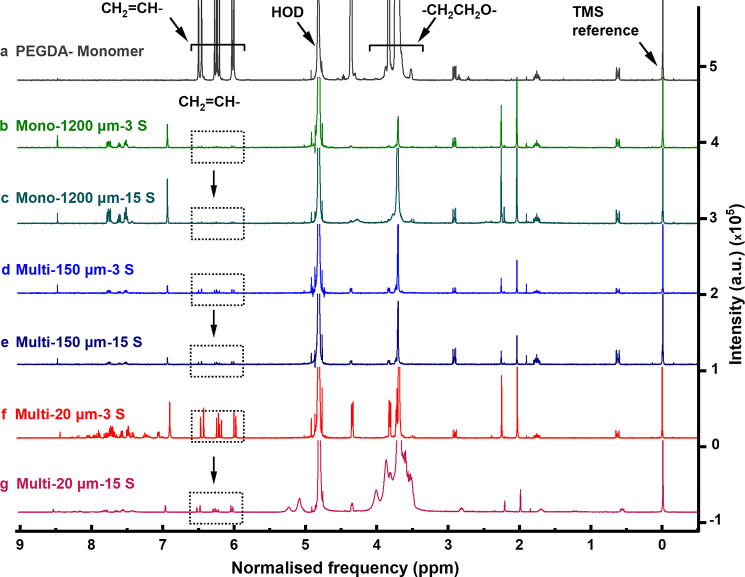
^1^H NMR spectra in D_2_O with a TMS reference peak at 0.00 ppm of samples. (a) PEGDA monomer showing the proton absorptions of the −CH_2_CH_2_O− and CH_2_=CH− groups centered at 3.80 and 6.25 ppm, respectively, with an intensity range of 0−5 × 10^5^. (b−g) Soluble fractions recovered from 3D PEGDA hydrogels stored for 24 h storage in D_2_O: (b, c) samples mono-1200 μm-3 s and mono-1200 μm-15 s, (d, e) samples multi-150 μm-3 s and multi-150 μm-15 s, and (f, g) samples multi-20 μm-3 s and multi-20 μm-15 s. The intensity range for all spectra is 0–5 × 10^5^.

To verify the degree of PEGDA cross-linking, ^1^H NMR analysis was used to assess the remaining solid residue recovered after fabrication from the hydrogels in D_2_O after swelling in water for 24 h. The ^1^H NMR spectra for PEGDA monomers show absorptions of protons of the −CH_2_CH_2_O– and CH_2_ = CH– groups centered at 3.80 and 6.25 ppm, respectively (Figure 6a). For all fabricated samples, the ^1^H NMR spectra (Figure 6b–g) show traces of unreacted PEGDA’s absorption centered at 3.80 and 6.25 ppm, which corresponds to protons of −CH_2_CH_2_O– and CH_2_ = CH– groups, respectively. The spectra also showed traces of absorption of the LAP photoinitiator at 2.00 and 2.25 ppm (CH_3_ groups), aromatic protons between 6.90 and 8.20 ppm, and QY photoabsorber aromatic protons between 6.90 and 8.20 ppm (Figure S2). These spectra confirm the presence of unreacted monomers that leach out of cross-linked hydrogels over time.

^1^H NMR analysis support the argument that an increased UV light dosage increases cross-linking in the 3D printed PEGDA structures. As previously reported,^20^ monolithic PEGDA hydrogel samples made in the absence of a QY photoabsorber had the highest degree of cross-linking, whereas multilayered PEGDA hydrogels, multi-150 μm-3 s and multi-20 μm-3 s, with 9 and 1.8 mg/mL of QY photoabsorber concentrations had lower cross-linking, respectively. In mono-1200 μm-3 s stored in D_2_O for 24 h and consequently analyzed by ^1^H NMR spectroscopy, evidence of traces of protons of −CH_2_CH_2_O– and CH_2_ = CH– groups centered at 3.80 and 6.25 ppm, respectively, was observed, confirming that the amount of unreacted prepolymers was negligible albeit not zero (Figure 6b). The peaks of protons of CH_2_ = CH– groups centered at 6.25 ppm were within the noise level for the mono-1200 μm-15 s (Figure 6c), which suggests that the fabrication of 3D monolithic samples with a prolonged UV exposure time of 15 s or 600 mJ/cm^2^ dosage yields the highest degree of cross-linking of the PEGDA prepolymer into PEGDA polymer among all other hydrogels. The increase of absorption centered at 3.8 ppm is due to the −CH_2_CH_2_O groups of PEGDA oligomers soluble in D_2_O.

In multi-20 μm-3 s and multi-150 μm-3 s made by exposure of 3 s of UV light, the ^1^H NMR shows a slightly higher absorption of the CH_2_ = CH– protons centered at 6.25 ppm with respect to the 3D monolithic, mono-1200 μm-3 s hydrogels (Figure 6d,f). This absorption reduced in 3D multilayered PEGDA hydrogels exposed to 15 s of UV light, confirming that longer exposure to UV light leads to a higher conversion of the unsaturated CH_2_ = CH– groups compared to saturated −CH_2_CH_2_O– groups, which means a higher degree of cross-linking in those hydrogels (Figure 6e,g). The UV light promotes the formation of more PEGDA oligomers soluble in D_2_O in the sample multi-20 μm-15 s, comprising 60 layers, than that in multi-150 μm-15 s, comprising eight layers. This suggests that the multi-150 μm-15 s hydrogels have a higher degree of cross-linking than the multi-20 μm-15 s. The ^1^H NMR results indicate that a targeted combination of UV light exposure and number of hydrogel layers has an inverse response to layer thickness, increasing the cross-linking in PEGDA hydrogels.

## 4 Discussion

The *E* obtained in this study agrees with those previously reported for PEGDA hydrogels with a similar molecular weight (Table S1). As the nanoindentation takes place on the topmost layer in a very shallow area with indentation depths varying between 0.5 and 2.7 μm, the measured *E* (Figure 3b) represents the material within the indentation and its immediate surroundings rather than the overall mechanical state of the bulk hydrogel (Figure 1).^51^ Such a shallow indentation depth was intentionally selected to minimize the influence of the hard substrate on *E*. For indentation depths shallower than 10% of the overall structure height, the substrate effect is practically negligible albeit not zero.^52,53^ Note that the indentation depth in this study is less than 3 μm on a 1200 μm thick structure (Figure 1). As the overall structure heights are the same for all samples, the magnitude of substrate effect is the same across all samples.

In terms of the relationship between *E* and the layer thickness, the nanoindentation measurements on 3D printed hydrogels do not agree with the typical “the thinner the stronger” behavior. The negative correlation between *E* and the number of layers may originate from the change in the QY photoabsorber concentration in the prepolymer solution (Figure 3b), which was 0 mg/mL for the mono-1200 μm-3 s, 1.8 mg/mL for multi-150 μm-3 s, and 9 mg/mL for the multi-20 μm-3 s. A previous study showed that the variation in the QY photoabsorber concentration affects the degree of cross-linking in the formed layers.^20^ A constant QY photoabsorber concentration at 1.8 mg/mL was therefore used to eliminate the effect of QY and to allow the assumption of the same degree of cross-linking for the multi-300 μm-3 s, multi-150 μm-3 s, and multi-37.5 μm-3 s samples (Figure 3d). Based on the initial argument that only the first few microns of the top hydrogel layer are affected by nanoindentation,^54^*E* is expected to be independent from the number of layers, as they are fabricated from the same chemical formulation. However, the measurements in Figure 3d show a 72% reduction in *E* when the number of layers increased by 8-fold from 4 to 32 layers. As described above, the degree of cross-linking positively affects the elastic modulus of hydrogel structures (Figures 3b and 5b–g). This agrees with the literature where 3D printed multilayered hydrogels showed lower compressive *E* in comparison with cast monolithic hydrogels.^46,55^

To minimize the creep effect on the measured *E*, a short holding time of 2 s was selected as suggested by the previous studies.^56−58^ However, some degree of creep deformations was still observed in the 3D printed PEGDA samples (Figure 4a). During the holding period at a constant load, the change in the displacement for multi-20 μm-3 s was observed in the range of 4–5% (Figure S3a), while for multi-150 μm-3 s and mono-1200 μm-3 s, it was 2–3%. This suggests that only multi-20 μm-3 s samples fall within the description of a quasi-creep condition, as a change in displacement in the range of 5–10% is required, while the other two fall into a noncreep condition.^58^ A longer holding time is required for future studies to fully establish the viscoelastic behavior of 3D printed PEGDA.^59,60^

Note that the curing time of 3 s, equivalent to 120 mJ/cm^2^ UV light dosage, was adopted from previous study where high resolution perfusable microchannels were needed.^61^ However, the optimization of the prepolymer solution composition has not covered the effect of different printing parameters on the degree of cross-linking of the 3D multilayered hydrogels. Here, the effect of prolonged UV exposure time on the nanomechanical response of the 3D hydrogels was tested by printing multilayered and monolithic hydrogels where each layer was exposed to 15 s, equivalent to 600 mJ/cm^2^ dosage of UV light. The exposure time of 15 s, which is half order of magnitude higher, was chosen to (i) achieve highly cross-linked printed layers, (ii) avoid excessive printing time, and (iii) prevent overexposure, which may lead to nonuniform layers due to over cross-linking.^62^

**Figure 7:**
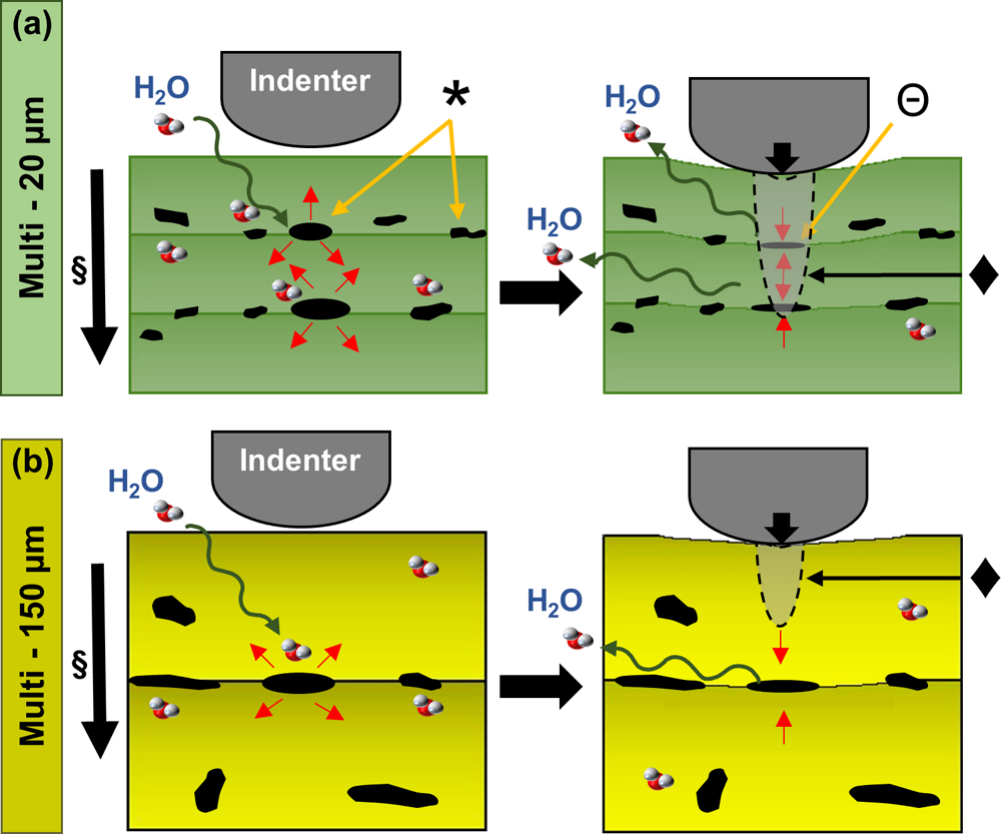
Schematic of the effect of the voids in the interface of the multilayered hydrogels (a) multi-20 μm and (b) multi-150 μm PEGDA hydrogels on the indentations and indenter displacement. Direction of projection light (§) is from top to bottom of the structure, (*) empty pockets of voids swollen due to water uptake, (Θ) collapsed pockets due to localized compression from the indenter, and (⧫) deformation zone of indentation.

Nanoindentation measurements along with SEM imaging and NMR analysis suggested that the surface nanomechanical properties of 3D printed PEGDA structures are highly dependent on the degree of cross-linking and defect density of the layers and their interfaces. Given that the indentation displacement occurs normal to the printed layers, the applied external force facilitates the collapse of pockets of voids in multiple layers and interfaces immediately beneath the indenter. The observed higher indentation depth (*h*_max_) of the multi-20 μm-3 s (Figure 7a) suggests that this mechanism is true for 3D printed PEGDA structures with low layer thickness. Thus, the overall nanomechanical response is a combination of response from the topmost layer, the immediate surrounding that includes the preceding printed layers, and the interfaces in between. In contrast, the lower *h*_max_ of multi-150 μm-3 s suggests that the collapse of voids is limited to only a few layers and interfaces in 3D printed PEGDA structures with low layer thickness, as they are further away from each other (Figure 7b). These findings imply that the nanomechanical properties of 3D printed hydrogel structures can be engineered by controlling the layer thickness or the number of layers without necessarily altering the chemical composition of the constituent materials and the UV curing dosage. Future investigation on single-layer PEGDA samples with different layer thicknesses is needed to further discriminate the influence of layer thickness from the number of layers on *E*.

## 5 Conclusions

Nanoindentation was performed to investigate the influence of the degree of cross-linking and the increased number of layers in multilayered PEGDA structures compared to a monolithic structure. The nanoindentation elastic modulus of multilayered PEGDA structures decreases with an increase in the number of layers. An increase in the layer number from 8 to 32 leads to a decrease in *E* by (−)218%. The layer-by-layer UV cross-linking process was observed to result in internal defects at the interfaces and within the cross-linked layer, where the defect density as voids increases with the increasing number of layers. Although far from the indentation location, these voids contribute to a reduced *E*. The creep index was also observed to increase with the increasing number of layers. An increase in UV light dosage from 3 s (120 mJ/cm^2^) to 15 s (600 mJ/cm^2^) leads to an increase in the degree of cross-linking that ultimately enhances the modulus by 96 and 84%, respectively. This is confirmed by ^1^H NMR spectroscopy where samples exposed to 15 s UV light showed a lower absorption of protons of CH_2_ = CH– groups, confirming a higher degree of cross-linking. Overall, monolithic samples can be considered as homogeneous structures with higher elastic modulus. In comparison, multilayered samples show heterogeneity and lower elastic modulus due to the interfacial defects, voids, unreacted prepolymers, and residues of the LAP photoinitiator and QY photoabsorber within the polymer network.^50^ The findings presented herein should be considered as a basis for further studies into fabrication parameters and structure design to enable precision engineering of 3D printed multilayered hydrogel structures, which will benefit future tissue engineering and organ-on-chip devices.

## Data Availability

Data underlying this study can be accessed through CORD at 10.17862/cranfield.rd.19390616.

## Supporting Information Available

Reported literature on PEGDA hydrogels; nanoindentation background on Oliver and Pharr method; explanations of force–displacement curve components; ^1^H NMR spectra for the LAP photoinitiator and QY photoabsorber; creep properties of PEGDA hydrogels; defect identifications and measurements; defect density calculations; gravimetric measurements explanations, and additional DSC measurements (PDF)
