# Supplementary material for: Nanoindentation Response of 3D Printed PEGDA Hydrogels in a Hydrated Environment
Source: ACS Appl Polym Mater. 2023 Jan 20;5(2):1180–90. doi: 10.1021/acsapm.2c01700 (PMC9926483; doi:10.1021/acsapm.2c01700)
Supplement: Supplementary file 1 — ap2c01700_si_001.pdf [file ap2c01700_si_001.pdf]

SUPPORTING INFORMATION

"Nanoindentation Response of 3D Printed PEGDA Hydrogels in a Hydrated Environment"

Mohammad Hakim Khalili [a], Craig J Williams [b], Christian Micallef [a], Fabian Duarte-Martinez [a], Ashfaq Afsar [c,d], Rujing Zhang [e], Sandra Wilson [e], Eleftheria Dossi [d], Susan A. Impey [a], Saurav Goel [f,g]*, and Adrianus Indrat Aria [a]*

[a] Surface Engineering and Precision Centre, School of Aerospace, Transport and Manufacturing, Cranfield University, Cranfield MK43 0AL, U.K.

[b] The Henry Royce Institute, Department of Materials, The University of Manchester, Manchester M13 9PL, U.K.

[c] School of Chemistry, University of Edinburgh, David Brewster Road, Edinburgh EH9 3FJ, U.K.

[d] Centre for Defence Chemistry, Cranfield University, Shrivenham, Swindon SN6 8LA, U.K.

[e] Sophion Bioscience A/S, Baltorpvej 154, 2750 Ballerup, Denmark

[f] London South Bank University, 103 Borough Road, London SE1 0AA, U.K.

[g] University of Petroleum and Energy Studies, Dehradun 248007, India

*** Corresponding author: a.i.aria@cranfield.ac.uk; ORCID: 0000-0002-6305-3906**

*** Corresponding author: goeLs@lsbu.ac.uk; ORCID: 0000-0002-8694-332X**

Reported elastic modulus of PEGDA in literature

Table S1. Reported *E* for PEGDA hydrogels with similar molecular weight using compression, tensile and indentation test methods.

MW (KDA)	Sample type	Type of modulus	E (MPa)	Test method	Ref
0.4	Monolithic	Elastic modulus	1.80-3.30	Nanoindentation	[1]
0.7	Multilayer	Shear modulus	0.40	Compression	[2]
0.7	Monolithic	Elastic modulus	3.00	Compression	[3]
0.4	Monolithic	Compressive	0.10-0.20	Compression	[4]
0.7	Monolithic	Elastic modulus	0.07	Tensile	[5]
0.7	Monolithic	Elastic modulus	2.66-2.94	AFM	[6]
0.7	Multilayer	Elastic modulus	0.67-2.03	Nanoindentation	This study
0.7	Monolithic	Elastic modulus	3.00-5.53	Nanoindentation	This study

Data underlying this study can be accessed through CORD at **https://doi.org/10.17862/cranfield.rd.19390616**.

Nanomechanical analysis with Oliver and Pharr method

Oliver and Pharr method was used for estimating the elastic modulus (E) of the material from the load-displacement (P-h) plot (Figure S1).[7,8] Oliver and Pharr method relies on calculating the projected contact area from a power-law fitted to the unloading curve of the P-h plot by assuming it as elastic.[9] This method relies on calculating the tip-sample contact size at the top 1/3rd part of unloading curve. The slope of the curve which measures the stiffness (S), through ${dP}/{dh}$ where (P) is the contact force or load and (h) is the relative movement of the centres of the spheres, enables one to obtain the reduced elastic modulus (Er) of the material based on the projected tip-sample contact area (A_c) through (eq S1 and S2),[10]

$$E_r = \frac{1}{\beta}\frac{\sqrt{\pi}}{2}\frac{S}{\sqrt{A_c}} \qquad \text{(eq S1)}$$

$$A_c = \pi a^2 \qquad \text{(eq S2)}$$

where β = 1 is a constant for the spherical indenter. The tip-shape function (a) for a spherical tip with a radius (R_i) (eq S3) can be obtained by measuring the contact depth (h_c) at maximum load (P_{max}) and maximum indenter displacement (h_{max}) using (eq S4)[11]

$$a = \sqrt{2R_i h_c - h_c^2} \qquad \text{(eq S3)}$$

$$h_c = h_{max} - k\frac{P_{max}}{S} \qquad \text{(eq S4)}$$

The (k) is a geometric constant that was reported to be $k = 0.75$ for spherical and paraboloid of revolution.[7]

Based on the calculated (Er), the elastic modulus (E) of the material can be obtained using (eq S5)[12]

$$\frac{1-\vartheta^2}{E} = \frac{1}{E_r} - \frac{1-\vartheta_{indenter}^2}{E_{indenter}} \qquad \text{(eq S5)}$$

where (ϑ) is Poisson's ratio. The equation can be simplified to:

$$E \approx (0.75) \times E_r \qquad \text{(eq S6)}$$

because the indenter tip is diamond which is much stiffer to *E* with several orders of magnitude higher than the hydrogels, thus the second term on the right-hand side of (eq S5) is negligible than the first term. By assuming the Poisson's ratio of the PEGDA hydrogel to be 0.5, because of the elastomeric character of its network,[1,13] *Er* can be multiplied by a constant of 0.75 to calculate *E* (eq S6).

Several assumptions were made before the data analysis. Firstly, the indentation behavior was assumed to be a sink-in type rather than pile-up around the indentation area. Secondly, the variation due to thermal drift was assumed to be negligible because the sample was kept in the same environment for 2 h before testing to make sure it has similar temperature as the tip and the enclosure parts. Additionally, each indentation run was set to 12 s in total which reduced risk of thermal drift during the measurement runs.

For calculating creep index C_{IT}:

$$C_{IT}(\%) = \frac{h_{max} - h_b}{h_{max}} \times 100 \qquad \text{(eq S7)}$$

$$\dot{\varepsilon} = \frac{1}{h_i}\left(\frac{dh}{dt}\right) \qquad \text{(eq S8)}$$

where (h_i) is the depth of the indenter at ith time step, ($\frac{dh}{dt}$) is the velocity of the indenter, (h_b) and (h_{max}) are the indentation depths at the beginning and end of the hold period, respectively.

Typical force-displacement curve during nanoindentation

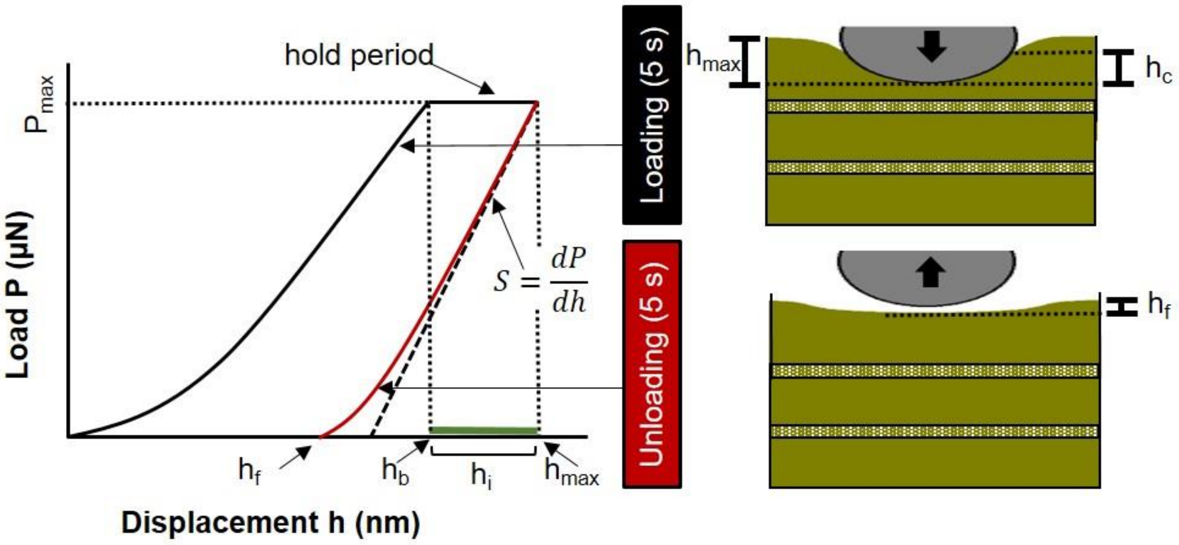

Figure S1. Schematic of typical (*P*-*h*) graph during nanoindentation with a hold period with graphical explanation of loading and unloading of a spherical indenter on a multilayered hydrogel sample. P_{max} is the maximum load just before unloading, S is the top 1/3rd unloading stiffness, h_f is the residual depth of nanoindentation, h_b is the indentation depths at the beginning of the hold period, h_{max} is the maximum depth of nanoindentation and h_i is the depth of the indenter at i_{th} time step during the hold period.

^{1}H NMR spectra for the LAP photoinitiator and the QY photoabsorber

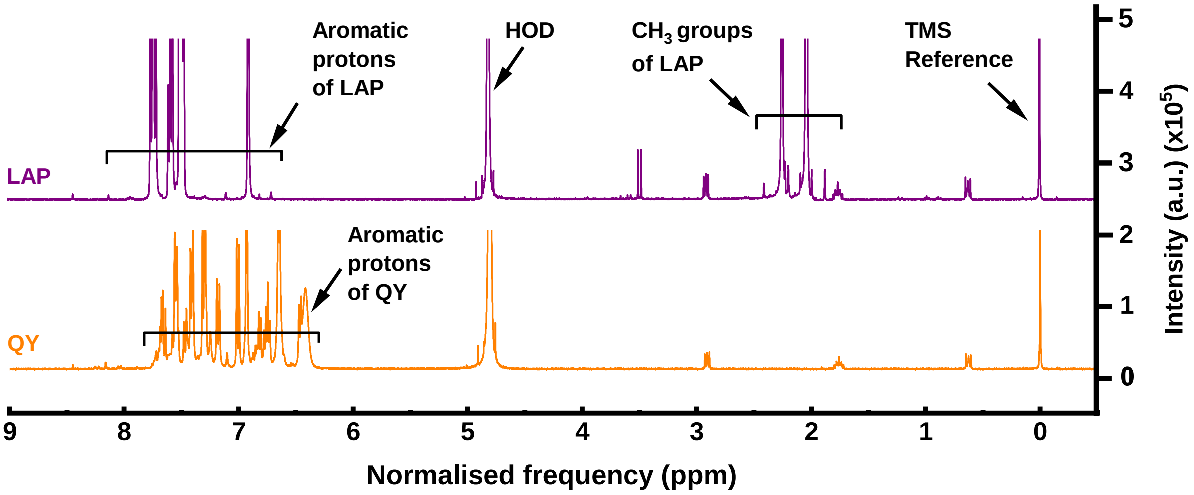

Figure S2. ^{1}H NMR spectra of the starting materials in D_2O. The LAP photoinitiator (top) exhibits peaks of CH_3 groups at 2.00 and 2.25 ppm and aromatic protons between 6.90 and 8.20 ppm. The QY photoabsorber (bottom) exhibits peaks of aromatic protons between 6.2 and 7.90 ppm. The peak at 0 ppm is attributed to TMS reference.

Detailed creep properties of PEGDA hydrogels

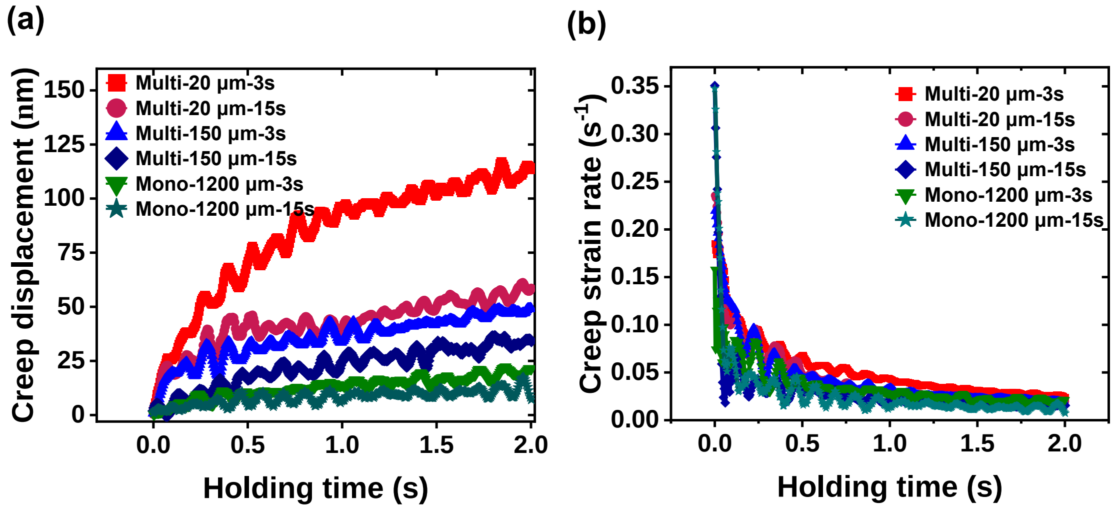

Figure S3. Nanoindentation creep properties; Representative (a) creep displacement curves during a constant load holding period, (b) creep strain rate at constant load of 20 µN, for the 3D multilayered, multi-20 µm-3 s (■), multi-20 µm-15 s (●), multi-150 µm-3 s (▲), and multi-150 µm-15 s (◆), and monolithic, mono-1200 µm-3 s (▼), and mono-1200 µm-15 s (★) PEGDA hydrogels after 24 h of storage in DIW post printing. Error bars represent standard deviation from a mean of n=3.

Defects identifications and measurements

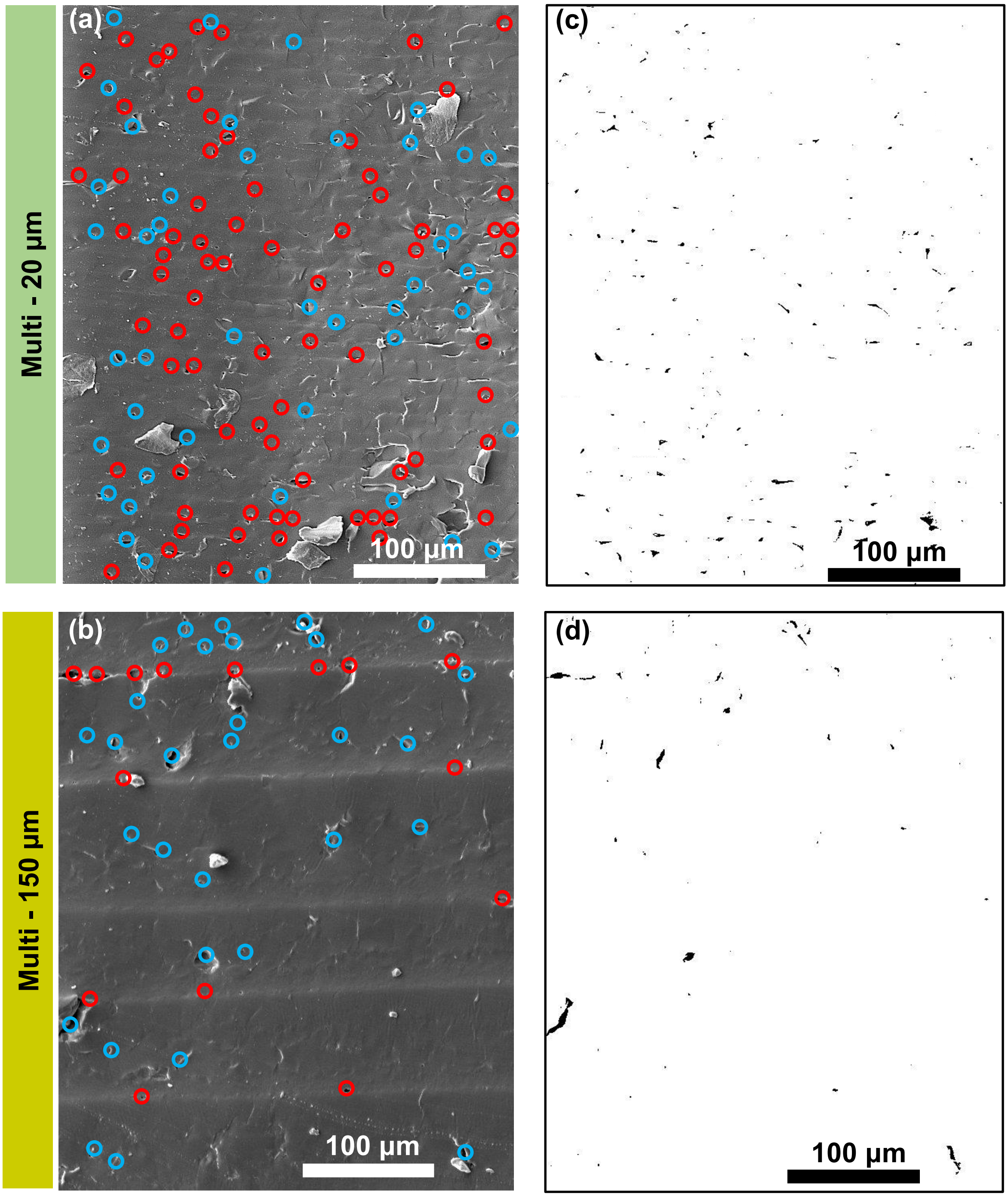

Figure S4. (a,b) SEM image of vacuum-dried multi-20 µm-3 s and multi-150 µm-3 s PEGDA hydrogels respectively showing all identified pockets of voids on the interface (red dots) and on the layer (light blue dots). Samples were washed and stored in DI water for 24 h prior vacuum-drying to purge any left unreacted prepolymers from the hydrogel. (c,d) Measurement of surface area of the identified pockets of voids using imageJ software.

Defect density calculations

Table S2. Calculation of defect density for (a) multi-20 µm-3 s and (b) multi-150 µm-3 s based on the pockets of voids at the interfaces and on the layers of the SEM images of vacuum-dried 3D PEGDA hydrogels.

**(a)**			
**Multi-20 µm-3 s**			
	S1	S2	Average
**Total area (µm^2)**	154,000	15400	
**Total apparent voids surface area (µm^2)**	4406	4930	
**Defect density (%)**	2.86	3.20	**3.03**

**(b)**			
**Multi-150 µm-3 s**			
	S1	S2	Average
**Total area (µm^2)**	242377	592901	
**Total apparent voids surface area (µm^2)**	1342	2052	
**Defect density (%)**	0.55	0.35	**0.45**

Gravimetric measurements

As the 3D PEGDA hydrogels are intended for use in biological application, their behavior in a relevant physiological environment were tested. Initially, the weight change NWF (eq 10) due to the temperature variations of the 3D multilayered hydrogels over the period 720 h (Figure 2), 30 days, was measured (Figure S5). For the 3D multilayered, multi-20 µm-3 s, PEGDA hydrogels stored at 8 and 20 °C in both DIW and CCM, there was a weight increase of between +15% to 20%, indicated by increase in NWF between 1.15 and 1.19, within the first 6 h of storage, respectively (Figure S5). The change in NWF slowed considerably after the first 24 and at 720 h, it has reached 1.16 and 1.20 for samples stored at 8 and 20 °C in DIW and CCM, respectively. The samples stored at 37 °C, both in CCM and DIW, had a subtle weight change over the same period of time with NWF of 1.02 and 1.03 at 24 h. However, their final weight at 720 h has increased to NWF of 1.05 and 1.07 in CCM and DIW, respectively (Figure S5). The multi-150 µm-3 s PEGDA hydrogels had a similar behavior to that of the multi-20 µm-3 s at 8 and 20 °C but the magnitude of the change was lower with reported NWF of 1.12 and 1.06 in CCM and DIW, respectively. However, the samples stored at 37 °C had almost 10% water loss in the first 6 to 24 h but over time they regained some of the weight loss and reached NWF of 0.96 and 0.94 when stored in CCM and DIW, respectively (Figure S5).

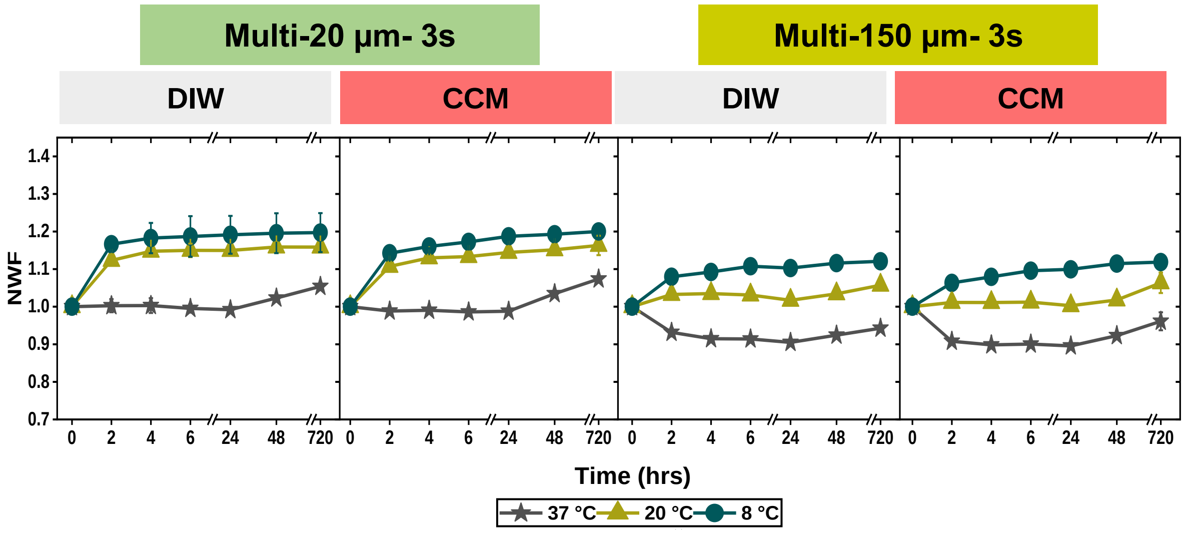

Figure S5. NWF of 3D multi-20 µm-3 s and multi-150 µm- 3s PEGDA hydrogel samples, both stored at 8 (●), 20 (▲) and 37 °C (★) in DIW and CCM for 720 h (30 days) measured at predetermined time points immediately after printing (0 h). Error bars represent standard deviation from the mean, with n = 2 for independent multilayered samples. Some error bars are not visible as they are smaller than the data point symbols. All lines are guide to the eye only.

Additional DSC measurements

The T_g of vacuum-dried multilayered PEGDA hydrogel samples, of multi-20 µm-3 s and multi-150 µm-3 s, at 24 and 720 h of storage in DIW and CCM, was recorded. Figure S6b, shows the thermograms of both type of samples and how their T_g increase of 3 °C, from -40 to -37 °C, when stored in DIW from 24 to 720 h. This can be due to the leaching out of more uncross-linked prepolymers from the polymer network and been replaced with DIW which increased the water content of the hydrogel and its purity which resulted in increased T_g.[14] The T_g of hydrogel samples which were stored in CCM did not increase of the same amount as the relative samples stored in DIW (Figure S6b). This could be due to the availability of various inorganic salts such as sodium bicarbonate in the CCM which either prevented i) the unreacted monomer to leach out from the polymer network and/or ii) the salts infiltrated in the polymer network resulting in higher T_g in comparison with samples stored in DIW.[15]

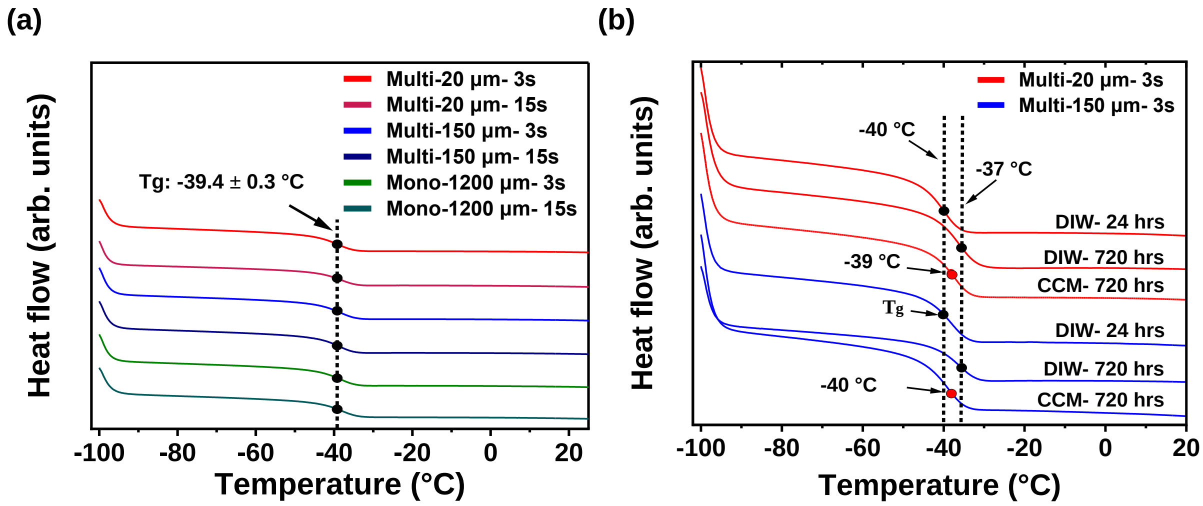

Figure S6. (a) Thermograms of 3D multilayered, multi-20 µm-3 s (▬), multi-20 µm-15 s (▬), multi-150 µm-3 s (▬), and multi-150 µm-15 s (▬) and monolithic, mono-1200 µm-3 s (▬), and mono-1200 µm-15 s (▬) PEGDA hydrogels stored in DIW for 24 h. (b) Thermograms of multi-20 µm-3 s (red, top three spectrum) and multi-150 µm-3 s (blue lines, bottom three spectrum) at 24 h and 720 h of storage in DIW of dry PEGDA hydrogels' second heating from -100 °C to 25 °C, at 10 °C min^{-1}.

References

(1) Gäbler, S.; Stampfl, J.; Koch, T.; Seidler, S.; Schüller, G.; Redl, H.; Juras, V.; Trattnig, S.; Weidisch, R. Determination of the Viscoelastic Properties of Hydrogels Based on Polyethylene Glycol Diacrylate (PEG-DA) and Human Articular Cartilage. *Int. J. Mater. Eng. Innov.* **2009**, *1* (1), 3–20. https://doi.org/10.1504/IJMATEI.2009.024024.

(2) Zhang, R.; Larsen, N. B. Stereolithographic Hydrogel Printing of 3D Culture Chips with Biofunctionalized Complex 3D Perfusion Networks. *Lab Chip* **2017**, *17* (24), 4273–4282. https://doi.org/10.1039/c7lc00926g.

(3) Yasar, O.; Inceoglu, S. Compressive Evaluation of Polyethylene (Glycol) Diacrylate (PEGDA) for Scaffold Fabrication. *ASME 2016 11th Int. Manuf. Sci. Eng. Conf. MSEC 2016* **2016**, *2*, 1–6. https://doi.org/10.1115/MSEC2016-8619.

(4) Mazzoccoli, J. P.; Feke, D. L.; Baskaran, H.; Pintauro, P. N. Mechanical and Cell Viability Properties of Crosslinked Low and High MW PEGDA Blends. *J. Biomed. Mater. Res. A* **2010**, *93* (2), 558–566. https://doi.org/10.1002/jbm.a.32563.Mechanical.

(5) Hockaday, L. A.; Kang, K. H.; Colangelo, N. W.; Cheung, P. Y. C.; Duan, B.; Malone, E.; Wu, J.; Girardi, L. N.; Bonassar, L. J.; Lipson, H.; Chu, C. C.; Butcher, J. T. Rapid 3D Printing of Anatomically Accurate and Mechanically Heterogeneous Aortic Valve Hydrogel Scaffolds. *Biofabrication* **2012**, *4* (3). https://doi.org/10.1088/1758-5082/4/3/035005.

(6) Drira, Z. Investigation of the Mechanical Properties of Poly(Ethylene Glycol) Diacrylate by Nanoindentation Using Atomic Force Microscopy, 2006. https://doi.org/10.1016/j.jmbbm.2012.09.015.

(7) Smallman, R. E.; Ngan, A. H. W. Characterization and Analysis. *Mod. Phys. Metall.*

2014, 159–250. https://doi.org/10.1016/b978-0-08-098204-5.00005-5.

(8) Oliver, W. C.; Pharr, G. M. An Improved Technique for Determining Hardness and Elastic Madulus Using Load and Displacement. *J Mater Res* **1992**, *7* (1), 1564–1583.

(9) Oliver, W. C.; Pharr, G. M. Measurement of Hardness and Elastic Modulus by Instrumented Indentation: Advances in Understanding and Refinements to Methodology. *J. Mater. Res.* **2004**, *19* (1), 3–20. https://doi.org/10.1557/jmr.2004.19.1.3.

(10) Pathak, S.; Kalidindi, S. R. Spherical Nanoindentation Stress-Strain Curves. *Mater. Sci. Eng. R Reports* **2015**, *91*, 1–36. https://doi.org/10.1016/j.mser.2015.02.001.

(11) Donohue, B. R.; Ambrus, A.; Kalidindi, S. R. Critical Evaluation of the Indentation Data Analyses Methods for the Extraction of Isotropic Uniaxial Mechanical Properties Using Finite Element Models. *Acta Mater.* **2012**, *60* (9), 3943–3952. https://doi.org/10.1016/j.actamat.2012.03.034.

(12) Goel, S.; Cross, G.; Stukowski, A.; Gamsjäger, E.; Beake, B.; Agrawal, A. Designing Nanoindentation Simulation Studies by Appropriate Indenter Choices: Case Study on Single Crystal Tungsten. *Comput. Mater. Sci.* **2018**, *152* (January), 196–210. https://doi.org/10.1016/j.commatsci.2018.04.044.

(13) Cappello, J.; d'Herbemont, V.; Lindner, A.; Roure, O. du. Microfluidic In-Situ Measurement of Poisson's Ratio of Hydrogels. *Micromachines* **2020**, *11* (3), 1–12. https://doi.org/10.3390/mi11030318.

(14) Ding, H. Y.; Li, Y.; Yang, G. N.; Yao, K. F.; Qiu, S. B. The Effect of Purification on the Glass-Forming Ability of a Pd-Cu-Si Alloy. *Metall. Mater. Trans. A Phys. Metall. Mater. Sci.* **2012**, *43* (8), 2610–2614. https://doi.org/10.1007/s11661-011-0812-y.

(15) Haruna, N.; Huang, J. Cytology & Tissue Biology Investigating the Dynamic Biophysical Properties of a Tunable Hydrogel for 3D Cell Culture. **2020**, 3–10. https://doi.org/10.24966/CTB-9107/100030.
